# Genome-Wide Expression of Transcriptomes and Their Co-Expression Pattern in Subtropical Maize (*Zea mays* L.) under Waterlogging Stress

**DOI:** 10.1371/journal.pone.0070433

**Published:** 2013-08-06

**Authors:** Nepolean Thirunavukkarasu, Firoz Hossain, Sweta Mohan, Kaliyugam Shiriga, Swati Mittal, Rinku Sharma, Rita Kumari Singh, Hari Shankar Gupta

**Affiliations:** Maize Genetics and Breeding Unit, Division of Genetics, Indian Agricultural Research Institute, Pusa, New Delhi, India; National Taiwan University, Taiwan

## Abstract

Waterlogging causes extensive damage to maize crops in tropical and subtropical regions. The identification of tolerance genes and their interactions at the molecular level will be helpful to engineer tolerant genotypes. A whole-genome transcriptome assay revealed the specific role of genes in response to waterlogging stress in susceptible and tolerant genotypes. Genes involved in the synthesis of ethylene and auxin, cell wall metabolism, activation of G-proteins and formation of aerenchyma and adventitious roots, were upregulated in the tolerant genotype. Many transcription factors, particularly ERFs, MYB, HSPs, MAPK, and LOB-domain protein were involved in regulation of these traits. Genes responsible for scavenging of ROS generated under stress were expressed along with those involved in carbohydrate metabolism. The physical locations of 21 genes expressed in the tolerant genotype were found to correspond with the marker intervals of known QTLs responsible for development of adaptive traits. Among the candidate genes, most showed synteny with genes of sorghum and foxtail millet. Co-expression analysis of 528 microarray samples including 16 samples from the present study generated seven functional modules each in the two genotypes, with differing characteristics. In the tolerant genotype, stress genes were co-expressed along with peroxidase and fermentation pathway genes.

## Introduction

In south and southeast Asia, approximately 18% of the land on which maize is grown is severely affected by floods; in India, 25%–30% of the production is lost annually for the same reason [1]. The plants wilt within a few hours to 2–4 days after exposure to flooding [2]. Stomatal closure and the humid environment lead to impaired root hydraulic conductivity [3]. Reduced edaphic components (Mn^2+^, Fe^2+^, S^2−^), volatile lower organic acids (propionic and butyric), and gases such as ammonia, carbon dioxide, ethylene, hydrogen sulfide, and methane [4] accumulate in waterlogged soils and damage roots. Reduced availability of oxygen in the rhizosphere severely constrains the plants’ capacity to produce ATP by mitochondrial oxidative phosphorylation, given that oxygen is the terminal electron acceptor [5]. Efficient uptake of such essential macronutrients as nitrogen, phosphorus, and potassium is affected by the reduced availability of ATP; carbohydrate reserves are depleted [6]; and the activities of enzymes that take part in the TCA cycle also decrease [7].

In response, plants employ alternative mechanisms such as glycolysis to generate ATP and ethanolic fermentation to produce the NAD^+^ required for sustaining the EMP pathway [8]. A set of anaerobic peptides including aldolase, enolase, glucose-6-phosphate isomerase, glyceraldehyde-3-phosphate dehydrogenase, sucrose synthase, and alcohol dehydrogenase have been identified as being selectively induced under hypoxia in maize [9]. The expression levels of a zinc finger-like protein, SKP1/ASK1-like protein, and 20S proteasome subunit α-3 increased markedly after 2 h of minimal oxygen supply [10]. A low oxygen-sensing N-end rule proteolytic pathway [11] and a gene, *Sicyp51*, believed to confer tolerance to hypoxia were identified recently [12]. SNORKEL1 and 2 in rice and MYB, and AP2/ERF transcription factors (RAP2.12, HRE1) in *Arabidopsis* [13]–[15] were found to regulate gene expression under low oxygen. Early studies in maize focused on the role of the ethylene-induced *XET* in aerenchyma formation in the cortical region of roots [16]. Calcium-dependent cysteine proteases have been implicated in the death of the maize primary root tip [17] and the G-box binding factor GBF1 in inducing *ADH1* promoter [18].

The availability of sequence information has facilitated research beyond transcriptomics toward proteomics and interactomics [19], [20]. Significant associations in the form of functional gene clusters may imply transcriptional coordination of genes [21]. The alignment of networks from different species has revealed evolutionarily conserved patterns [22]. The co-expression network approach has identified candidate genes for glucosinolate accumulation in *Arabidopsis* [23] while expediting the process of discovery of new genes [24]. The approach can retrieve information about genes with functions that are not known yet, whereas differential co-expression analysis can uncover the steps involved in metabolic pathways [25].

In the present study, a whole-genome transcriptome assay was performed at three stages of waterlogging stress in subtropical maize genotypes to (1) study the expression pattern of transcriptomes in genotypes tolerant and susceptible to waterlogging, and identify the roles of differentially expressed genes (DEGs) in important pathways that underlie the adaptive traits; (2) co-map the bin locations of the transcriptomes with already known QTLs for waterlogging and find synteny with other species; (3) generate gene co-expression networks to explore cohorts of genes expressed together in modules and functional clusters, while comparing the two contrasting genotypes.

## Materials and Methods

### Stress Treatment

**Figure 1:**
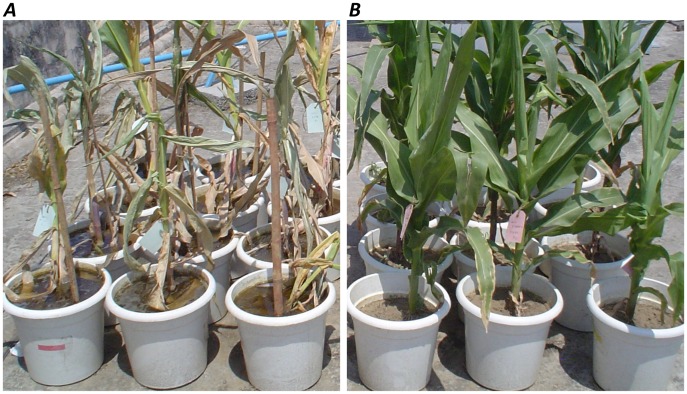
Response of genotypes to control and severe stages of the waterlogging stress. (*A*) Represents severe stress stage of HKI 1105 (first two rows) and V 372 (last two rows), and (*B*) Represents control stage of HKI 1105 (first two rows) and V 372 (last two rows).

Two subtropical maize (*Zea mays* L.) inbred lines, HKI 1105 (tolerant to waterlogging stress) and V 372 (susceptible to waterlogging stress), were sown in 35 cm tall plastic pots filled with sandy loam soil (Figure 1). The plants were watered daily to field capacity until the 28th day after sowing, after which they were subjected to stress, in the form of waterlogging, for a standardized duration of seven days, based on the observation that seven days of waterlogging causes adequate stress to maize plants [26]–[28]. Waterlogging was ensured by sealing the drainage holes at the bottom of the pots and maintaining 5 cm of standing water. Root samples were collected on 28, 32 and 35 days after sowing, which represented the control, moderate stress and severe stress, respectively. To allow the plants to recover from stress, the seal was removed on the 42^nd^ day after sowing (post stress recovery) so that excess water could drain out freely and subsequently root sample was collected from the recovered plants.

### Isolation, Labeling, and Hybridization of RNA

Total RNA was isolated and purified from root samples (50 mg each) using the RNeasy mini kit (Qiagen, Hilden, North Rhine-Westphalia, Germany) after grinding the tissues in liquid nitrogen, following the manufacturer’s guidelines. Total RNA was checked for quantity and quality using a NanoDrop 1000 spectrophotometer (Thermo Scientific, Wilmington, Delaware, USA) and denaturing agarose gel electrophoresis, respectively. Affymetrix GeneChip Maize Genome Array (Affymetrix Inc., Santa Clara, California, USA) representing 13339 genes was used for the microarray experiment. Approximately 300 ng of total RNA was biotin-labeled for GeneChip analysis and 10 µg of purified fragmented cRNA was used for hybridization. Hybridization, washing, and scanning were performed as described in the GeneChip standard protocol (3′-IVT Express kit user’s manual). Two technical replicates of each sample were taken to test both the reproducibility and quality of chip hybridization.

### Microarray Normalization and Data Analysis

Through the GeneChip operating software (GCOS, Affymetrix GeneChip operating software with autoloader, ver. 1.4, manual), CEL files were generated after scanning. The data was submitted to the NCBI GEO (Gene Expression Omnibus) (www.ncbi.nlm.nih.gov/geo) database (accession # GSE43088). The raw CEL files containing probe intensities from 16 chips were imported into the R platform using *affy* package [29]. The GeneChip Robust Multiarray Average (GCRMA) algorithm was used for background correction, normalization, and probe set summarization [30]. Linear modeling of microarray data and identification of DEGs was performed with *limma* package [31]. It computes moderated t-statistics and log-odds of differential expression by empirical Bayes shrinkage of the standard errors toward a common value. Probe sets having a *p* value of ≤0.001 and ≥fivefold change were considered differentially expressed under waterlogging with respect to the control and were computationally annotated using Blast2GO 1.3.3 [32]. Only plant-specific annotations were taken into account. Pathway visualization for moderate and severe stress stage transcripts was performed using MapMan [33].

### Construction of Co-expression Networks

Co-expression networks were created using 511 samples obtained from NCBI GEO, 64 from EBI ArrayExpress Archive, and 16 from in-house waterlogging microarray data (platform accession number GPL4032, Table S1 in File S1). All 591 samples were RMA-normalized with the *affy* package, and outliers were identified using three statistical tests provided by the Bioconductor package arrayQualityMetrics. Sixty-three samples failed at least one test and were discarded, and the remaining 528 samples were considered for network construction. To generate a co-expression network specific to waterlogging, the data subsets were restricted to genes that were differentially expressed in the in-house microarray experimental data. The differentially expressed probe sets were mapped to maize loci using maize B73 (ver. 5b.60) gene models (www.maizesequence.org). Probe sets that matched multiple genes as well as those that were redundantly mapped to a single gene were removed, and the retained probe sets were uniquely mapped to maize genes.

Finally, for network analysis, two data subsets were generated: for DEGs from the tolerant and from the susceptible genotype. Pairwise Pearson correlations were calculated for DEGs across all the samples to generate similarity matrices for the subsets. Next, the similarity matrices were converted to adjacency matrices using weighted gene correlation network analysis (WGCNA) by raising them to the power (β) that best approximates scale-free behavior of the resultant networks. The values for the soft threshold (β) were 10 and 16 for the subsets for the tolerant and susceptible genotypes, respectively. Topological overlap matrix (TOM) similarities were calculated from the adjacency matrices and were then used to calculate consensus dissimilarity. The latter was used as the input in average-linkage hierarchical clustering, and the minimum module size was set to 30. A dynamic tree cut algorithm was used to identify the branches of the resulting dendrogram. The eigengenes in each of the data subsets were calculated to determine whether some of the initial consensus modules should be merged. A “minimum consensus similarity” matrix was calculated as the minimum of the dataset eigengene correlation matrices, which was expressed as dissimilarity by subtraction from one and used as the input for average-linkage hierarchical clustering. In the resulting dendrogram, the modules on branches with a merging height of <0.2 were merged. Such branches corresponded to modules having eigengenes with a correlation of 0.8 or higher. This procedure finally produced seven consensus modules for each data subset. For each network module, a functional enrichment test based on Fisher’s exact test (*p* value <0.1) was performed against the genome background using DAVID online [34]. The modules were subdivided into functional clusters using pairwise *k* statistics between all genes.

### Colocalization with QTLs

The bin locations of 662 genes that were expressed exclusively during stress (but not during recovery) in the tolerant genotype were retrieved by querying against the maize genome database (www.maizegdb.org). The locations of the genes were then matched against QTLs of aerenchyma and adventitious root formation. A cross-species sequence similarity analysis was performed across five additional species: sorghum (http://www.phytozome.net/cgi-bin/gbrowse/sorghum/), foxtail millet (http://www.phytozome.net/cgi-bin/gbrowse/foxtail/), Brachypodium (http://www.phytozome.net/cgi-bin/gbrowse/brachy/), rice (http://www.plexdb.org/modules/MGI/), and *Arabidopsis* (http://www.plexdb.org/modules/MGI/) to identify the synteny of the colocalized genes.

### qRT-PCR Analysis

The microarray expression data were validated using two-step qRT-PCR (Agilent Technologies, Santa Clara, California, USA). First-strand cDNA was synthesized from 250 ng of total RNA using an Affinity Script qRT-PCR cDNA synthesis kit (Stratagene, Agilent Technologies). With the help of IDT software (http://eu.idtdna.com), gene-specific primers were designed (Table S2 in File S1). The subsequent reaction was performed using Stratagene MX3005P (Agilent Technologies). The conditions were as follows: 10 min at 95°C (preheating), followed by 40 cycles of amplification with denaturation for 30 s at 60°C, primer annealing for 1 min at 58°C, and primer extension for 30 s at 72°C.

### Root Section Analysis

Fresh root segments were collected on the sampling days (days 28, 32, 35, and 42 after sowing). Thin sections were prepared without mounting in wax [35]. The sections were observed through a Leica M205FA microscope (Leica Microsystems, Wetzlar, Hesse, Germany). The images were captured with an inbuilt camera (DFC425C).

## Results

### Gene Expression

**Figure 2:**
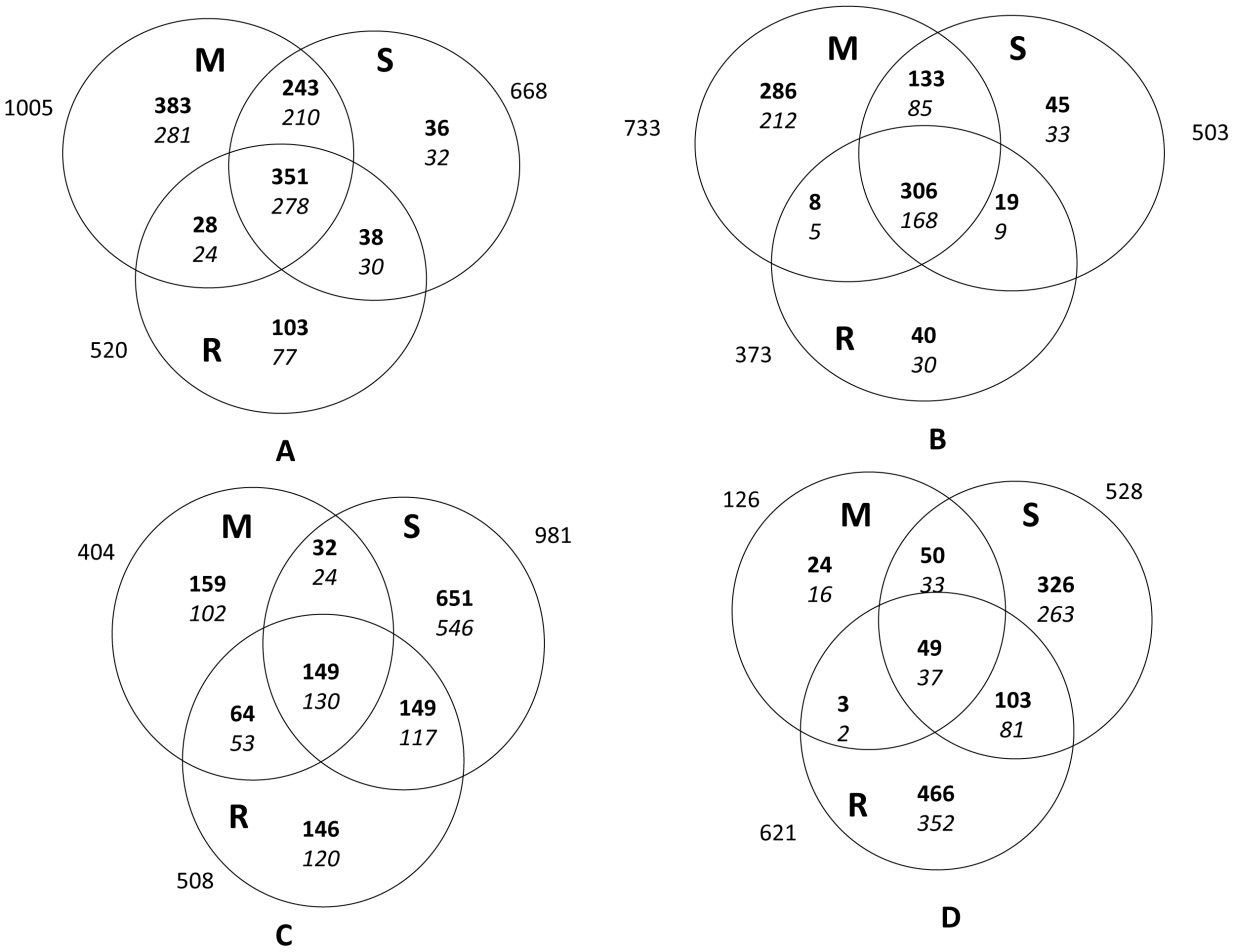
An overview of the differentially expressed genes (DEGs) at *p* ≤ 0.001 and ≥ fivefold expression at moderate (M), severe (S) and recovery (R) stages of waterlogging stress. The total number of DEGs is shown in bold. The number of genes having cellular component, molecular function, and biological process GO terms is shown in italics. (*A*) and (*B*) represent genes up and downregulated, respectively in the tolerant genotype (HKI 1105), whereas (*C*) and (*D*) represent those in the susceptible genotype (V 372).

The extent of hybridization of the cDNA with probe sets on the chip was 67%−75%. Genes with statistically significant differential expression in response to waterlogging and post-stress recovery were identified using R. Although the *p* value to be set as a threshold for accepting DEGs was ≤0.001, analysis revealed that the number of genes obtained at this level was close to the average number of genes obtained at ≤0.0001 and ≤0.01 (Table S3 in File S1). Of the 14850 transcripts interrogated by probe sets on the maize GeneChip, 8% (1188) were upregulated and 6% (891) were downregulated in HKI 1105 (tolerant), whereas 9% (1337) were upregulated and 7% (1040) were downregulated in V 372 (susceptible). However, the total number of genes obtained by summing the number of genes expressed at each stage was higher in HKI 1105. Comparison of the two genotypes at each stage revealed that when the stress was moderate, only 17% of the genes were differentially regulated in V 372 compared to 46% in HKI 1105 (Figure 2).

### Gene Annotation

Gene ontology (GO) terms, namely cellular component, molecular function, and biological process, were assigned to the BLAST hits of input sequences by Blast2GO (Tables S4*A*, S5*A*, S6*A* and S7*A* in File S1). Of the upregulated genes during severe stress, 82% (maximum in the tolerant genotype) were annotated, whereas 79% each of those expressed during moderate stress and during the recovery phase were labeled with a GO description. The GO terms were then grouped according to the MIPS functional catalogue. “Protein with binding function or co-factor requirement (structural/catalytic)” had the maximum representation (57%−61%). Other than “binding,” more specific GO terms such as “catalytic activity” (22%) and “nucleotide binding” (20%) were the best represented in the abovementioned MIPS category for genes upregulated in stress stages in HKI 1105. In the same category, in V 372, “oxygen binding” and “structural molecule activity” accounted for less than 1%. The categories “biogenesis” and “cell cycle” did not include downregulated genes.

**Figure 3:**
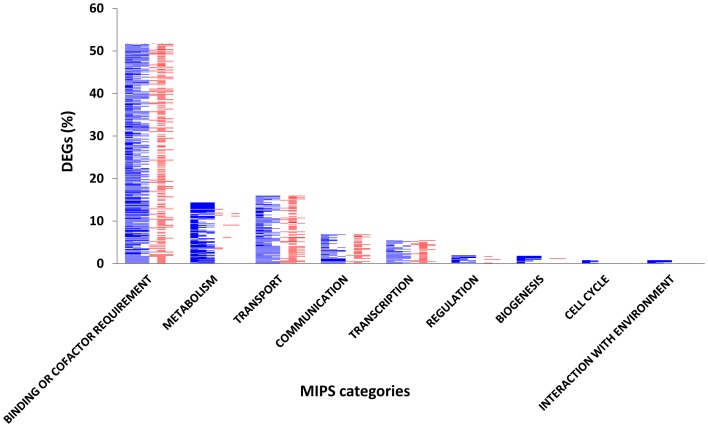
Blast2GO-annotated DEGs grouped according to the MIPS functional catalogue. The gene expression pattern across moderate stress, severe stress and recovery is shown in the form of heat maps. Blue represents the percentage of upregulated genes in HKI 1105 (tolerant genotype) while red represents downregulated genes in V 372 (susceptible genotype).

In HKI 1105, 13% and 11% upregulated genes accounted for metabolic and cellular transport functions, respectively, whereas 8% and 15% of genes, respectively, were downregulated in V 372 (Figure 3). In the latter, four classes, namely, “transport,” “cellular communication,” “metabolism,” and “transcription” each accounted for more than 5% of the downregulated genes.

### Analysis of Pathways and Transcript Levels

**Figure 4:**
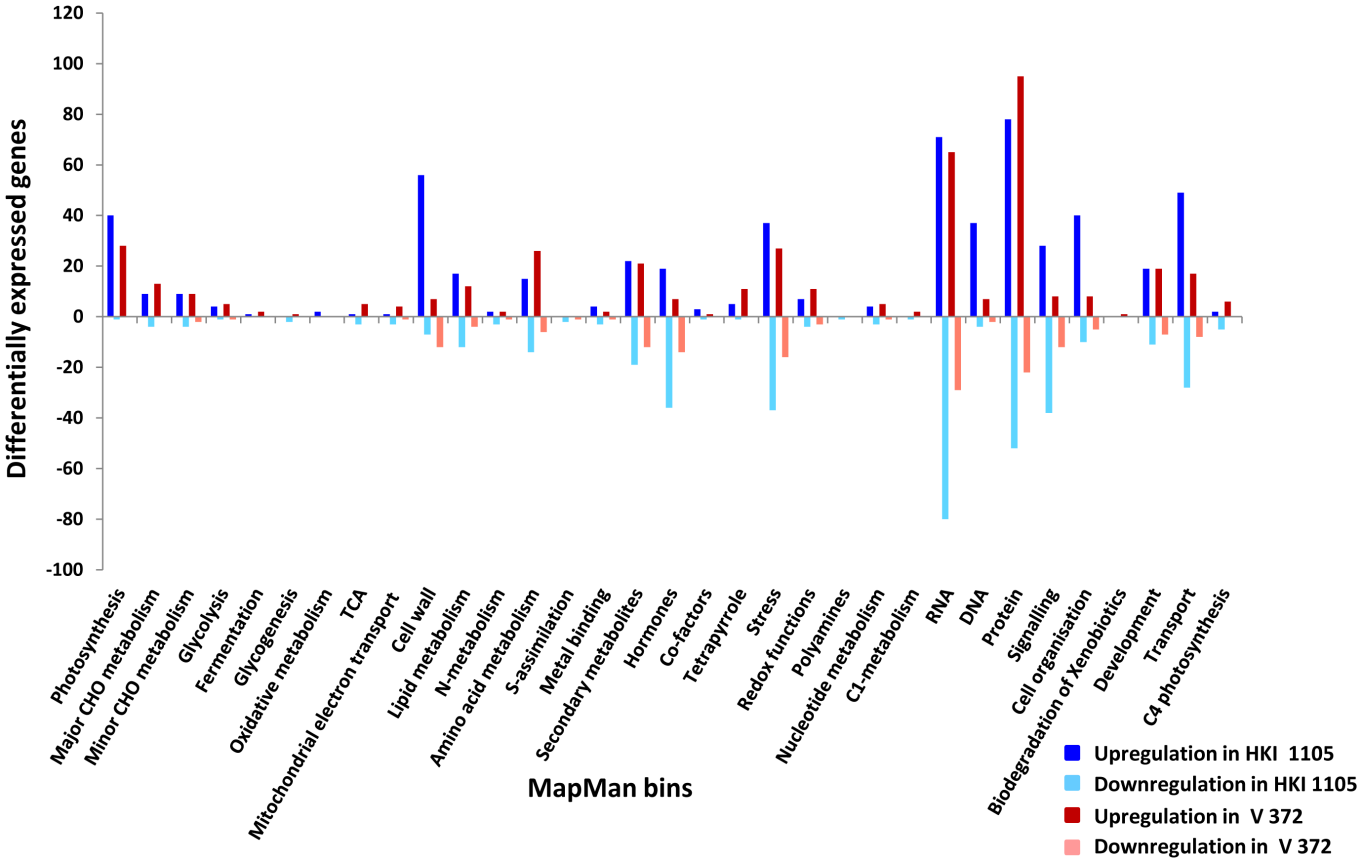
Representation of DEGs during waterlogging stress in various pathways according to MapMan. Dark blue and dark red represent number of upregulated genes while light blue and light red represent number of downregulated genes in HKI 1105 (tolerant genotype) and V 372 (susceptible genotype), respectively.

The analysis of the waterlogging-induced transcriptome data with MapMan provided information about the pathways in which the various DEGs were functionally important (Figure 4). In HKI 1105, of 1865 stress stage genes, 1532 were mapped to various bins. Nearly 74% genes were mapped to pathways, whereas the rest were unassigned. In V 372, 84.7% of 1150 genes were mapped to various bins, a percentage comparable to that of genes mapped in the tolerant genotype. Compared with HKI 1105, fewer than one-fifth the number of genes were mapped to “DNA” and “cell wall” in V 372.

The transcript levels of DEGs at the different stages were compared to identify the patterns of expression. For 72 genes that were upregulated in the tolerant genotype, the fold change value was >100% during both moderate and severe stress over the stage of recovery, indicating that the genes played major roles under stress conditions. Upon restoration of normal conditions, the transcript levels decreased, although for 45 genes the fold change value increased upon recovery. Approximately 30% of 1005 upregulated genes in HKI 1105 were expressed only under moderate stress and 5% were only expressed under severe stress. Of the genes expressed during severe stress, 36% were already induced in response to decreased oxygen during moderate stress. In contrast, in V 372, the number of genes uniquely induced during moderate stress was almost one-fourth of that induced during severe stress.

Cortical cell delineating protein precursor had the highest (3103-fold) and the second highest level of expression (1267-fold) during moderate and severe stress, respectively, in HKI 1105 (Table S4*A* in File S1). Highly induced or repressed genes that have not been described or annotated may be considered for future analysis (Tables S4*B*, S5*B*, S6*B* and S7*B* in File S1).

### Co-expression Networks

**Figure 5:**
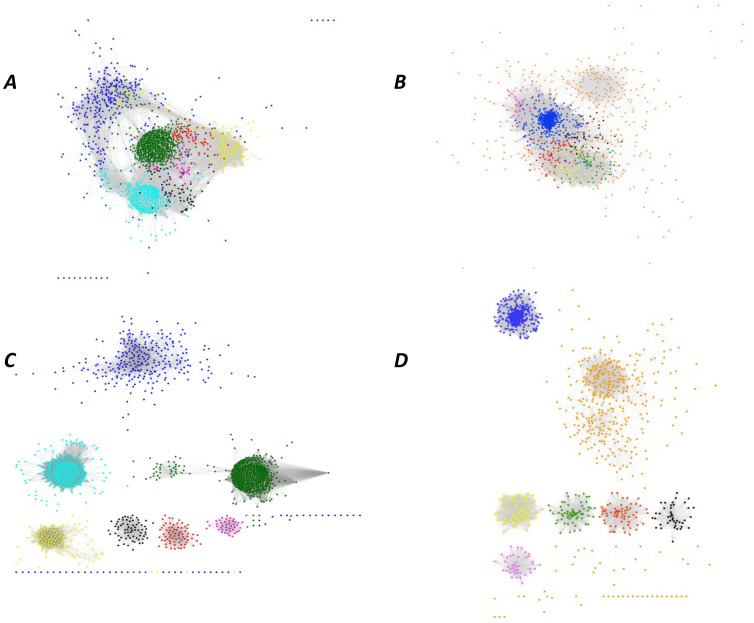
Co-expression network of genes involved in response to moderate and severe waterlogging stress. The network comprises 1593 nodes and 178,449 edges in HKI 1105, and 1538 nodes and 367,958 edges in V 372. Each color represents a module. (*A*) and (*C*) represent overviews of the co-expression networks in HKI 1105 and V 372 genotypes, respectively. (*B*) and (*D*) show the seven modules each in the tolerant and susceptible genotypes, respectively, extracted from the corresponding networks. (*A*) and (*B*): black, module 1; green, 2; blue, 3; magenta, 4; red, 5; turquoise, 6; yellow, 7; (*C*) and (*D*): black, 1; blue, 2; green, 3; orange, 4; pink, 5; red, 6; yellow, 7.

The co-expression network analysis was based on transcriptome metadata collected from NCBI GEO, EBI ArrayExpress Archive (Table S1 in File S1), and an in-house microarray experiment. The similarity matrices generated from 528 filtered samples were further processed to generate weighted co-expression network with scale-free topology by raising them to power β (Figure S1). For the tolerant and susceptible genotypes, 1654 and 1591 DEGs, respectively, were mapped to gene models (Table S8 in File S1). Network construction for the tolerant and susceptible genotype subsets yielded 1593 and 1538 nodes, connected by 178449 and 367958 edges, respectively. The global networks were further clustered into seven modules each in the two genotypes using WGCNA (Figure 5). Eigenvectors for the modules were calculated to evaluate the relatedness among the modules (Figure S2).

**Figure 6:**
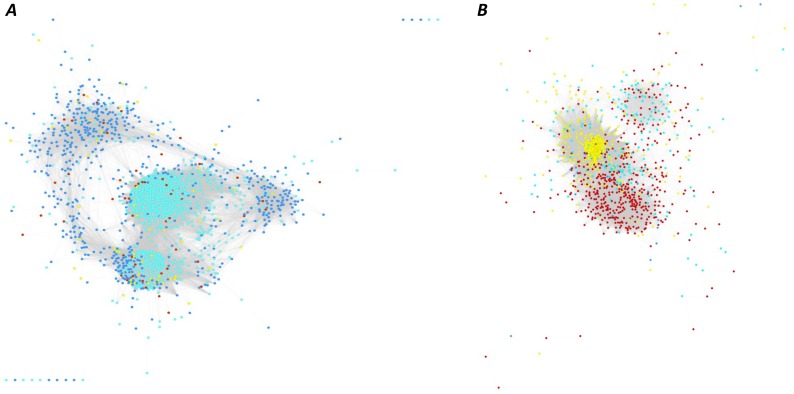
Differentially expressed genes mapped for stress conditions. Co-expression networks of the HKI 1105 and V 372 genotypes in (*A*) and (*B*) respectively, color-coded according to the level or state of stress and the nature of regulation. Moderate stress: upregulation, turquoise; downregulation, blue; severe stress: upregulation, yellow; downregulation, red.

**Figure 7:**
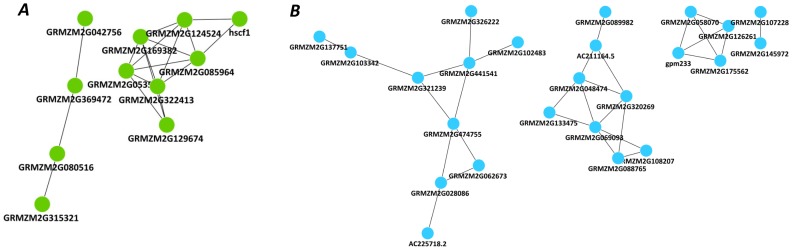
Functional clusters of genes identified in the tolerant genotype. Genes coding for (*A*) ethylene-responsive proteins, including AP2 domain-containing protein (GRMZM2G369472), ERF-like 1 (GRMZM2G053503), and EREBP 2 (GRMZM2G085964), and (*B*) Ca-binding proteins, including EF-HAND Ca-binding protein CCD1 (AC225718.2), calcineurin B-like protein 4 (GRMZM2G137751), calcium-dependent protein kinase isoform AK1 (GRMZM2G028086), and calmodulin (GRMZM2G062673) in HKI 1105.

In each genotype, the networks were also analyzed according to the differential regulation of genes under moderate and severe stress (Figure 6). Only modules 3 and 7 comprised >50% of downregulated genes in HKI 1105. The largest module consisted of 583 genes in HKI 1105 and 825 in V 372. The modules were divided into 229 functional clusters using pairwise *k* statistics between all genes. These functional clusters were further filtered based on Fisher’s exact test (*p*<0.1), which yielded 59 and 65 functional clusters for the tolerant and susceptible genotype networks, respectively (Table S9 in File S1). The stress-related cluster comprised 63 genes in HKI 1105 but only 45 in V 372. In HKI 1105, the ethylene-response factors and calcium-dependent protein kinases were enriched (Figure 7).

### Colocalization with QTLs

The bin locations of 662 genes that were expressed during stress (but not during recovery) in HKI 1105 were retrieved by querying against the maize genome database (www.maizegdb.org). Bin locations could be determined for 98% of the genes, of which the locations of 88 matched those of mapped QTLs of aerenchyma and adventitious root formation. On the basis of known markers in these bin locations, 21 genes were localized in marker intervals. Twenty genes co-localized with QTL of aerenchyma formation on chromosomes 2, 5, 8, and 9. Under moderate stress, the low-molecular weight cysteine-rich protein LCR69 precursor expressed the most (265-fold); the temperature-induced lipocalin expressed 52-fold; and an unknown gene co-located in the same bin location expressed 46-fold.

A cross-species sequence similarity analysis was performed across five additional species: sorghum, foxtail millet, rice, Brachypodium, and *Arabidopsis* to characterize the synteny of colocalized genes (Table S10 in File S1). Five genes, namely, temperature-induced lipocalin, potassium channel beta subunit, cytosolic orthophosphate dikinase, beta-tubulin 4, and chlorophyll a/b binding protein 2 were mapped across all the five species. All genes but one could be mapped in sorghum and 20 in foxtail millet, indicating that the two are very closely related to maize. The corresponding figures for the other three species were as follows: rice, 15; Brachypodium, 14; *Arabidopsis*, 5.

## Discussion

### Transcriptomic Expression for Adaptive Traits

**Figure 8:**
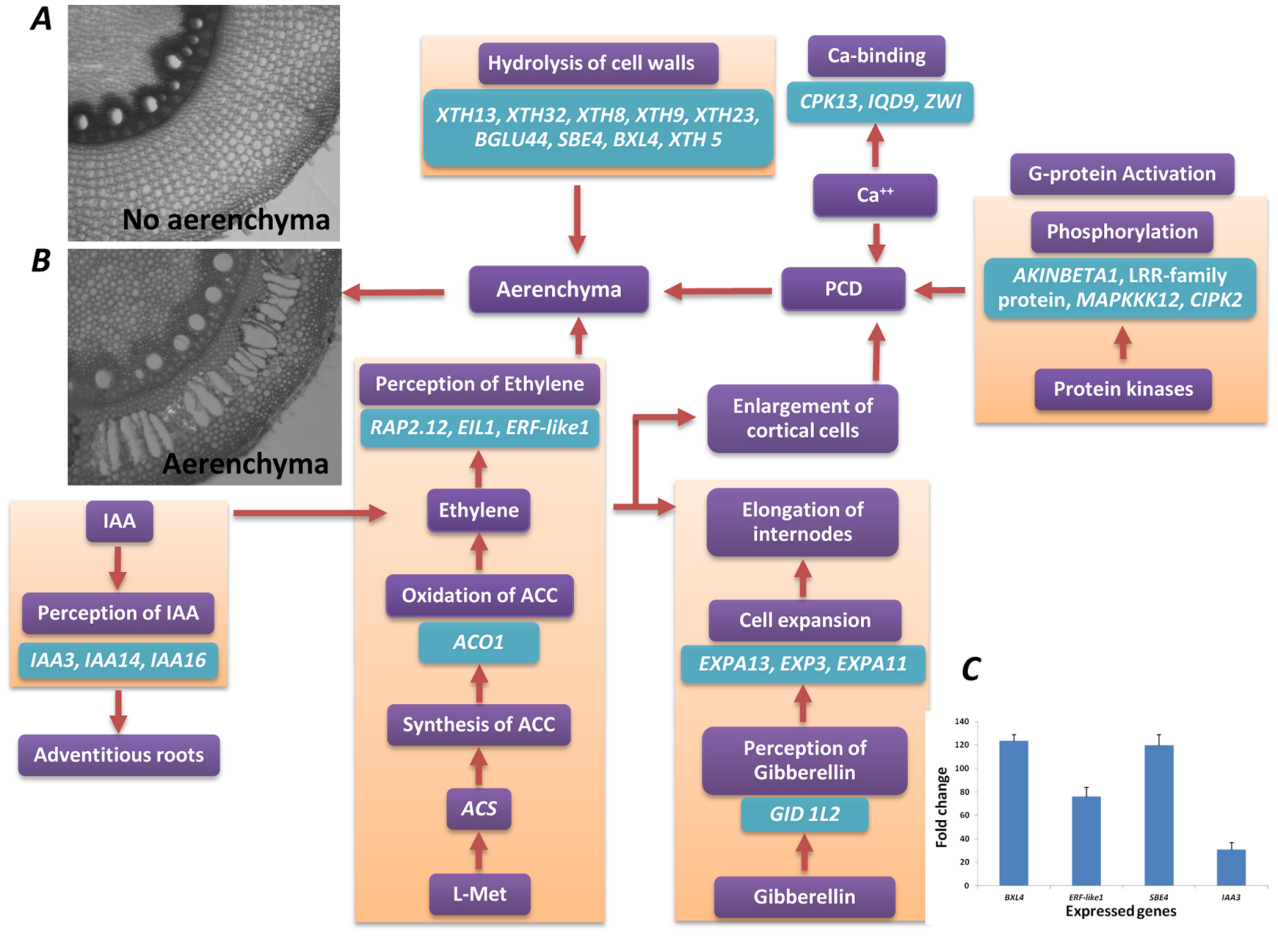
Combination of events and interactions during hypoxia, leading to programmed cell death (PCD). (*A*) No formation of aerenchyma at control stage in the absence of PCD and (*B*) formation of aerenchyma at severe stress stage due to PCD in the tolerant genotype (HKI 1105). (*C*) The differences in fold change of a few genes are represented with standard error in microarray and qRT-PCR assays.

Formation of aerenchyma is one of the mechanisms for adapting to stress by promoting gaseous exchange between roots and shoots. Aerenchyma is formed as a result of apoptosis or programmed cell death (PCD) in the root cortex [16]. PCD is triggered by a series of steps involving G-proteins and protein kinases, hydrolysis of cell walls, Ca-binding proteins, and ethylene and IAA synthesis during stress conditions (Figure 8). Studies of the signal transduction cascade have revealed that activation of G-proteins and protein kinases, inhibition of phosphatases, and increase in Ca^2+^ ion levels, all promote apoptosis [36]. All three features of PCD were observed in HKI 1105 (tolerant) exposed to stress in the form of waterlogging. During waterlogging, the cytoplasm becomes acidic, leading to the release of Ca^2+^ from mitochondria owing to loss of potential by the mitochondrial membrane [37]. Calmodulin, a Ca-binding protein that was expressed only in HKI 1105 and not in V 372, interacts with glutamate decarboxylase and helps to maintain cytosolic pH under anoxia [37].

Hydrolases, enzymes that degrade the cell wall, play an important role in anoxia-induced cell death and the formation of aerenchyma [38]. The actively dividing cells in the root tip consume much energy. To conserve energy as well as materials (substrate) to prolong survival, the actively dividing cells die as an adaptation to anoxia. A member of a flooding-specific gene family, *XET A* was found upregulated in HKI 1105 during both moderate (253-fold) and severe (16-fold) stresses, but downregulated in V 372. Expansins are a family of cell-wall proteins that loosen the cell wall by disrupting the cellulose-hemicellulose network at lower pH [39] resulting from hypoxia, and facilitate free sliding of the polymers over each other; upon restoration of normal conditions, the cell wall eventually regains its original structure [40]. A precursor of the alpha-l-fucosidase 2 gene, which helps to degrade glycan structures, was upregulated during moderate (134-fold) and severe (49-fold) stress in HKI 1105 and not expressed in V 372. Although it was also upregulated during the recovery stage in the tolerant genotype, its expression level was only about one-tenth of that achieved during the stress, probably because the breakdown of cell wall polymers is no longer required once hypoxia is overcome.

The plant hormones ethylene and IAA stimulate the development of adventitious roots, which replace basal roots as the latter become incapable of conducting water and nutrients under a reduced supply of oxygen [41]. Ethylene biosynthesis genes were downregulated in the susceptible genotype, whereas a number of genes encoding ethylene-binding proteins (AP2/EREBP family) such as ethylene-responsive factor-like protein 1, *BBM2*, *AIL5-like*, and *WRI1*, were upregulated in HKI 1105. It was also observed that in the tolerant genotype, auxin receptor genes such as, *IAA3*, *IAA14* and *IAA16* were up-regulated. An association between ethylene and auxin-signaling pathways is possible, given that ethylene enhances the formation of lateral and adventitious roots [42].

### Genes Involved in Metabolism and Assimilation of Energy

Abiotic stress affects CO_2_ diffusion, ribulose-1, 5-bisphosphate (RuBP) content (dependent on ATP and NADPH supply), RUBISCO activity, and photorespiration [43]. In HKI 1105, following exposure to stress, photosynthetic genes coding for pigment chlorophyll a/b binding protein-1 (13-fold) and -2 (34-fold), *CP24* (32-fold), *CP29* (28-fold), and *CP26* apoprotein precursor (1360-fold) were upregulated to keep photosynthetic efficiency as high as possible. Usually, anoxia results in inhibition of uptake of water through roots however few studies have reported the up-regulation of certain aquaporin genes, such as *NIP* [44], [45]. The latter was notably induced (116-fold in moderate stress, 76-fold in severe stress) in the present study too, in the tolerant genotype. Among the genes involved in carbohydrate metabolism, the gene that encodes the invertase enzyme also plays a key role in tolerance to hypoxia by mediating break down of starch and degradation of sucrose. Two other enzymes–a soluble acid invertase and orthophosphate dikinase, an enzyme of the glyoxylate cycle–were activated in HKI 1105 under stress. Lipid metabolism genes coding for desaturases (74-fold), LTPs (72-fold), esterases (21-fold), and lipases (26-fold) were upregulated. A plausible explanation is that lipid metabolism intermediates are converted to carbohydrates during stress [46]. These genes were neither upregulated nor downregulated in V 372. Fermentation is an important pathway operating under anoxia and enables the much-needed regeneration of NAD^+^ from NADH. The alanine fermentation pathway helps in regulating the pH of the cytoplasm under anoxia [47], ionic balance being a key component of adaptation to stress.

### Genes Involved in Regulation

Evidence gathered over the years suggests that transcriptional regulation plays a key role in helping the plant cope with hypoxia [48]. MYB transcription factors are known to trigger *ADH* [13], and WRKY transcription factors play a role in the plant’s response to biotic and abiotic stress, several developmental processes, and in senescence [49]. Several transcription factors such as the zinc-finger protein, CCCH-type family protein, auxin-responsive *AUX IAA* family member protein, homeobox, HSP, and MADS-BOX were upregulated in HKI 1105 (tolerant) as well as in *Arabidopsis* [50], whereas homeobox genes were down-regulated in V 372 (susceptible). The gene coding for LOB-domain proteins, which are plant-specific transcription factors involved in forming lateral roots [51], and responding to auxin [52], was significantly expressed in HKI 1105.

### Detoxification and Genes Involved in Abiotic Stress

GSTs, which play an important role in the plant’s response to various types of biotic and abiotic stresses [53], were activated only in HKI 1105. In V 372, peroxidase 1 precursor and peroxidase 72-like were downregulated, whereas in HKI 1105, peroxidase 72-like and peroxidase 2 genes were upregulated under moderate stress. Under anoxia and heat stress, plants produce H_2_O_2_ [54], which stimulates heat-shock proteins belonging to DNAJ-type HSPs and small HSP family. An increase in *HSP* transcripts in response to low oxygen has been observed across various kingdoms [55]. Activation of ROP (Rho-related GTPases from plants) through an NADPH oxidase mechanism leads to ROS accumulation in HKI 1105, and acts as a stimulus for *ADH* expression [56].

### Identification of Functional Clusters of Genes through Co-expression Networks

The gene co-expression networks behaved as biological networks found in nature; they were “small-world” (average path length of 1), scale-free, modular, and hierarchical. A negative linear correlation between the number of edges, log(*k*), and the probability of finding a node with *k* edges, P(*k*), indicated scale-free behavior (Figure S2). The plots showing the dependency of the clustering co-efficient on connectivity (Figure S2) and the module eigenvector clustering dendrogram (Figure S3) indicated hierarchical and modular behavior. The average clustering co-efficients were 0.167 and 0.198 for the tolerant and susceptible genotypes, respectively.

GO enrichment analyses for all modules were performed to identify the modules responsive to waterlogging in HKI 1105 (Table S11 in File S1). In module 2, the largest module, genes with “response to abiotic stimulus,” “cellular macromolecular complex assembly,” “GST activity,” “hydrolase activity,” and “XET activity” were co-expressed. Similarly, in module 6, *ACO1*, fermentation genes *AlaAT* and *PDC*, and photosynthesis genes, were co-expressed. This cofunctional saturation of molecular function terms in a tightly co-expressed gene module suggested an association between functional entities represented by the genes (such as protein domains and GO terms) and the phenotype under stress conditions. Moreover, the phenotypic association may be extended to the neighboring co-expressed genes whose sequence descriptions are yet to be reported. Thus, the selected gene sets become candidates for the underlying expression of the trait and provide hints at molecular pathways associated with the expression of the defined phenotypes. For instance, the first cluster of module 1 of tolerant genotype contained 18 genes, mostly with regulatory functions. The genes included those coding for ERF-like protein 1 and EREBP1 (which respond to ethylene), the transcription factor CCCH, and HMG1/2-like protein (the CoA-bound form of which is a stress-responsive protein). Thus, through “guilt by association,” seven hypothetical proteins are implicated in regulating various pathways under stress.

**Figure 9:**
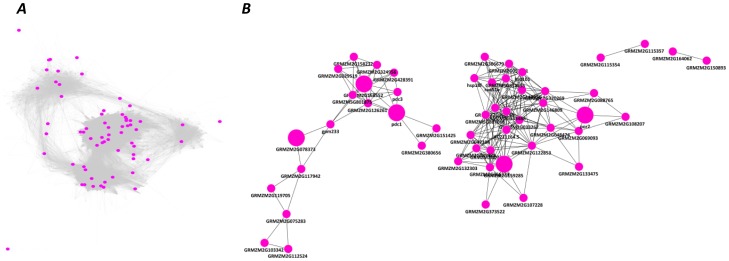
Stress tolerant gene clusters. Genes in the “response to stress” cluster in HKI 1105, shown (*A*) as part of the co-expression network. The nodes representing genes that play a crucial role in tolerance to waterlogging are shown magnified in (*B*). These include GRMZM2G078373: ASR protein, GRMZM2G168552: bundle sheath strand-specific gene 1, *PDC1,* GRMZM2G159285: *IAA13,* and *POR2.*

The “response to stress” gene cluster in HKI 1105 (Figure 9) included genes (*IAA13* and *IAA24*) coding for auxin response, crucial for the formation of adventitious roots. *PDC1,3*, the genes coding for the enzyme involved in converting pyruvate to acetaldehyde, and peroxidases involved in scavenging free radicals were present in the same cluster. Other stress-related genes found in various co-expressed clusters of HKI 1105 included plant proteinase inhibitors implicated in stress-induced PCD in plants [57], abscisic acid stress ripening gene (*ASR*), bundle sheath strand specific gene1 (*BSS1*), and universal stress protein (*USP*). Although *ASR* has no specific role in ripening, studies of melon have shown the presence of an ethylene-response element [58]. *BSS1* is highly homologous to the tomato *ASR1* in the C-terminal region and although its exact function is unknown, it may also be involved in stress response. USPs, expressed under hypoxia and anoxia [59], were activated under moderate stress (12-fold). In rice, they were found to be positively regulated under submergence [59]. In the “response to stress or stimuli” functional cluster of V 372, many of the abovementioned genes were absent. ERFs regulating aerenchyma formation were found in another cluster of HKI 1105. Other functional clusters comprised expansins, transport proteins, and EF-HAND proteins. In contrast, in V 372, the GO terms pertaining to aquaporins, expansins, Ca-binding proteins, AP2-domain proteins, and *XET*s were not enriched.

### Overlaying Transcriptomic Data with Mapped QTLs

Integration of transcriptional profiling with QTL analysis has been used for studying genes related to complex traits [60] in order to elucidate the gene–phenotype relationships. Yet, QTL mapping data have seldom been linked with microarray analysis on a genome-wide scale, particularly in maize exposed to waterlogging. Integrating the transcriptomes significantly expressed in HKI 1105, the tolerant genotype, with the reported QTLs provided clues to possible influences of some of the genes on expression of quantitative traits in response to waterlogging stress. Cytosolic orthophosphate dikinase and beta-tubulin 4 are probably most closely associated with the formation of adventitious roots, and calmodulin-binding protein and expansins colocalized with the QTL of aerenchyma formation. The same trait at bin 2.06 co-located with mitogen-activated protein kinase and a gene coding for histone protein. Histones of some genes have been found to be targets of reversible modifications under submergence conditions [61].

**Figure 10:**
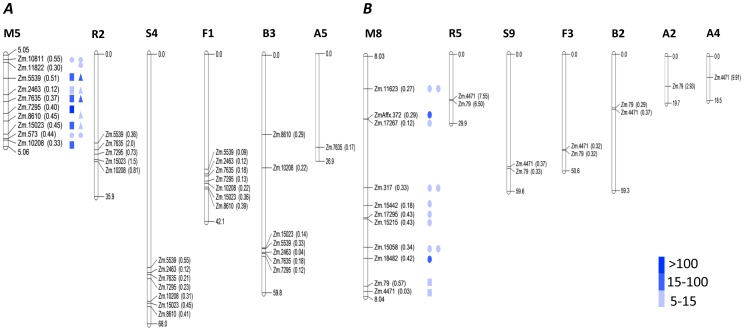
Co-localization of DEGs with known QTLs. Physical location (in parenthesis) of maize DEGs in HKI 1105 mapped on the marker intervals of known QTLs and the extent of synteny for (*A*) aerenchyma between bins 5.05 and 5.06 and for (*B*) adventitious roots between bins 8.03 and 8.04. The letters M, R, S, F, B, and A stand for the crops maize, rice, sorghum, foxtail millet, Brachypodium, and *Arabidopsis*, respectively. The number next to the letter represents the chromosome number. The first column of symbols indicates moderate stress and the second denotes severe stress. The expression levels of genes co-mapped in other crops are denoted by squares and triangles. Circle represents the DEGs not co-localized in any other crop.

The orthology of candidate genes was found in other crop species as well, of which the maximum number of genes was co-mapped in sorghum and foxtail millet. The bin location 5.05–5.06 was the most widespread, with seven genes showing orthology with other crops. In contrast, bin 8.03 was highly conserved, with only two genes showing such orthology (Figure 10). These observations suggest that phylogenetically, some genomic regions were favored during evolution, given that they occurred in multiple species, whereas others remained highly specific to maize.

### Conclusion

Low oxygen leads to extensive reprogramming of gene expression to help the plant withstand stress as well as to maintain photosynthesis and metabolism at optimum levels. The number of genes whose expression was repressed under waterlogging was lower than that of those that were activated, suggesting that under stress, amplifying rather than inhibiting the expression of a majority of genes could be a strategy for survival. The actions of expansins, hydrolases, cellulases, kinases, and the phytohormones ethylene and auxin complemented each other in the establishment of adaptive features. Transcriptional coordination of genes points to functionally significant associations such as the inclusion of stress and fermentation pathway genes in the same module in the tolerant genotype. Given that many of these “unknown” genes had high transcript levels under different levels of stress, their contribution to stress tolerance could be important. Colocalization with QTLs mapped in response to waterlogging opens up the possibility of using the induced genes as candidates for introgression into susceptible lines. The combined approach will also be relevant to future investigations on functional analysis of candidate genes.

## Supporting Information

Figure S1 **The analysis of network topology for various soft-threshold powers for the tolerant genotype.** (*A*) Shows the scale-free fit index (Y-axis) as a function of the soft-threshold power (X-axis) and (*B*) displays the mean connectivity (degree; Y-axis) as a function of the soft-threshold power (X-axis).
(TIF)

Figure S2 (*A*) and (*B*) Clustering of module eigenvectors (ME) (or first principal components) of co-expression networks comprising seven modules each in two genotypes. WGCNA was used for calculation of the eigenvectors and clustering. The closeness of modules implies similarity in expression patterns. The red line indicates a cut-off height of 0.2, corresponding to a correlation of 0.8. (*C*) and (*D*) Scale-free network topology indicated by a negative correlation between the number of edges [log(*k*)] and the probability of a node having *k* edges [P(k)]. (*E*) and (*F*) The graph of connectivity Vs clustering co-efficient, showing cliquishness of genes or modular behavior of the network.
(TIF)

Figure S3 A network heatmap depicting a topological overlap matrix (TOM) among all genes in the analysis in (*A*) tolerant and (*B*) susceptible genotypes. Light color represents low overlap and progressively darker red color represents higher overlaps. Blocks of darker colors along the diagonal are the modules, which are also represented as colored bars on the left vertical and top horizontal axes. Genes integrated into no module are shown in grey. The gene dendrograms are shown on top of the axes.
(TIF)

File S1 Table S1. Microarray samples of Platform: GPL4032 used in co-expression network construction. Table S2. Details of primers designed for qRT-PCR of selected genes. Table S3. The number of genes filtered at different *p* values is shown. *p* ≤ 0.001 was finally chosen as the cutoff for differential gene expression analysis. Table S4. Highly upregulated genes at moderate and severe stress stages in HKI 1105 (tolerant genotype). (*A*) Genes identified by sequence description and GO terms assigned through Blast2GO. (*B*) Genes not having uniquely identifying sequence description. Some of them were assigned GO terms. Table S5. Highly downregulated genes at moderate and severe stress stages in HKI 1105 (tolerant genotype). (*A*) Genes identified by sequence description and GO terms assigned through Blast2GO. (*B*) Genes not having uniquely identifying sequence description. Some of them were assigned GO terms. Table S6. Highly upregulated genes at moderate and severe stress stages in V 372 (susceptible genotype). (*A*) Genes identified by sequence description and GO terms assigned through Blast2GO. (*B*) Genes not having uniquely identifying sequence description. Some of them were assigned GO terms. Table S7. Highly downregulated genes at moderate and severe stress stages in V 372 (susceptible genotype). (*A*) Genes identified by sequence description and GO terms assigned through Blast2GO. (*B*) Genes not having uniquely identifying sequence description. Some of them were assigned GO terms. Table S8. Gene models of the (*A*) tolerant genotype and (*B*) susceptible genotype. Table S9. Functional clustering of co-expression network in (*A*) tolerant genotype and (*B*) susceptible genotype. Table S10. List of genes mapped with waterlogging QTLs in maize. The synteny of these genes with rice, sorghum, foxtail millet, Brachypodium, and *Arabidopsis* was also studied. Table S11. Important functional clusters in various co-expression network modules of tolerant genotype.
(XLSX)
