# Supplementary figures and images for: Genome-Wide Expression of Transcriptomes and Their Co-Expression Pattern in Subtropical Maize (Zea mays L.) under Waterlogging Stress
Source: PLoS One. 2013 Aug 6;8(8):e70433. doi: 10.1371/journal.pone.0070433 (PMC3735631; doi:10.1371/journal.pone.0070433)

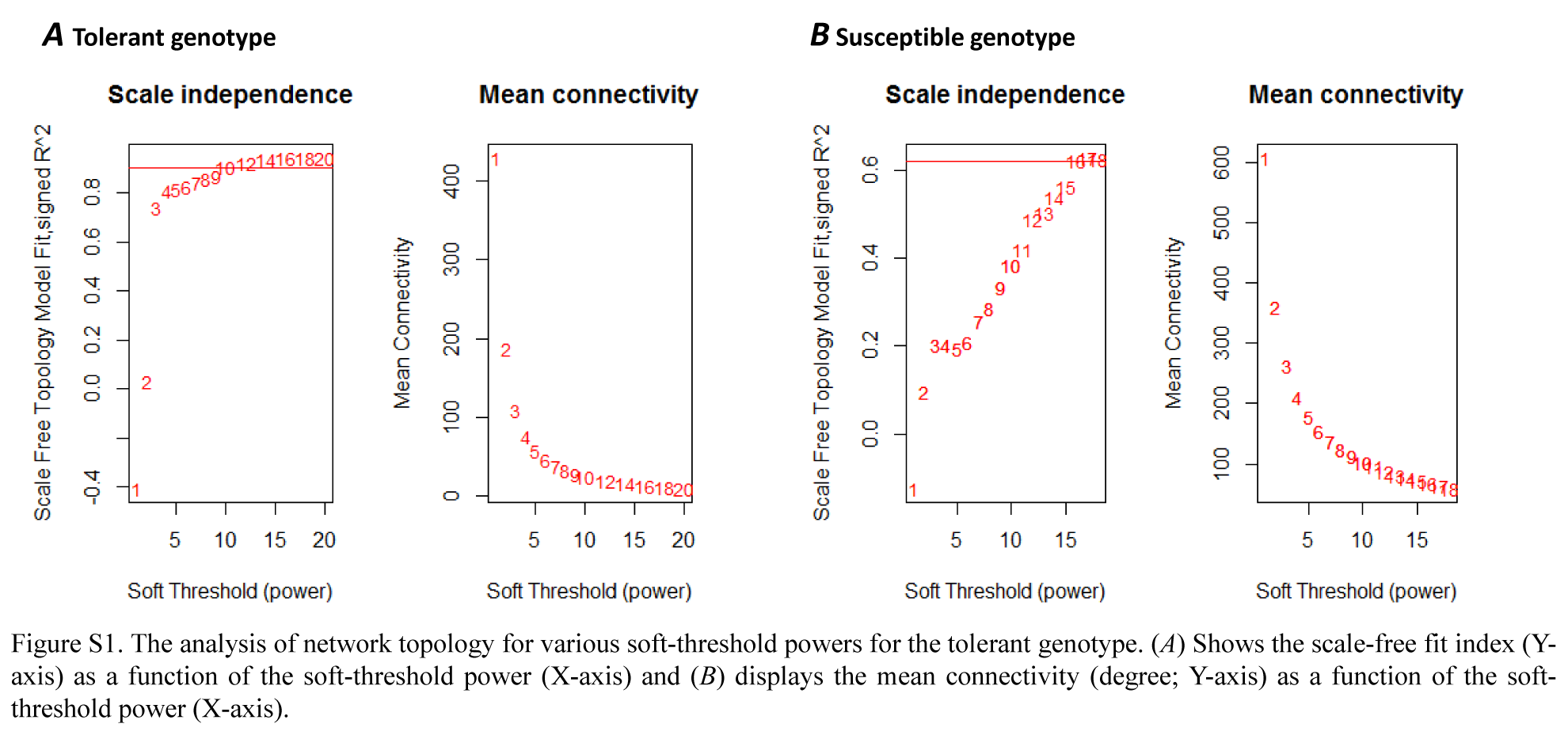

Supplement: Figure S1 — The analysis of network topology for various soft-threshold powers for the tolerant genotype. (A) Shows the scale-free fit index (Y-axis) as a function of the soft-threshold power (X-axis) and (B) displays the mean connectivity (degree; Y-axis) as a function of the soft-threshold power (X-axis). (TIF) [file pone.0070433.s001.tif]

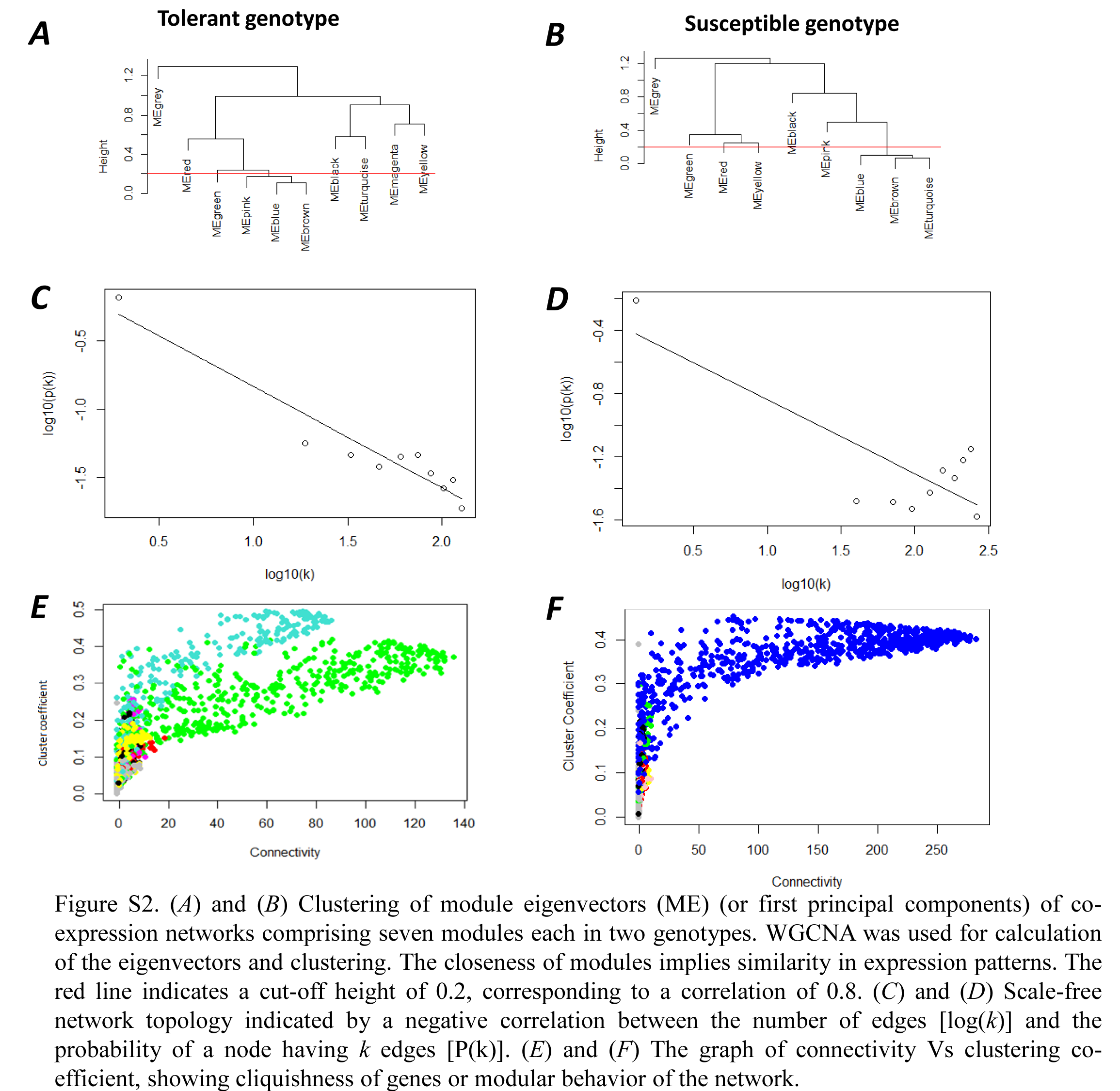

Supplement: Figure S2 — (A) and (B) Clustering of module eigenvectors (ME) (or first principal components) of co-expression networks comprising seven modules each in two genotypes. WGCNA was used for calculation of the eigenvectors and clustering. The closeness of modules implies similarity in expression patterns. The red line indicates a cut-off height of 0.2, corresponding to a correlation of 0.8. (C) and (D) Scale-free network topology indicated by a negative correlation between the number of edges [log(k)] and the probability of a node having k edges [P(k)]. (E) and (F) The graph of connectivity Vs clustering co-efficient, showing cliquishness of genes or modular behavior of the network. (TIF) [file pone.0070433.s002.tif]

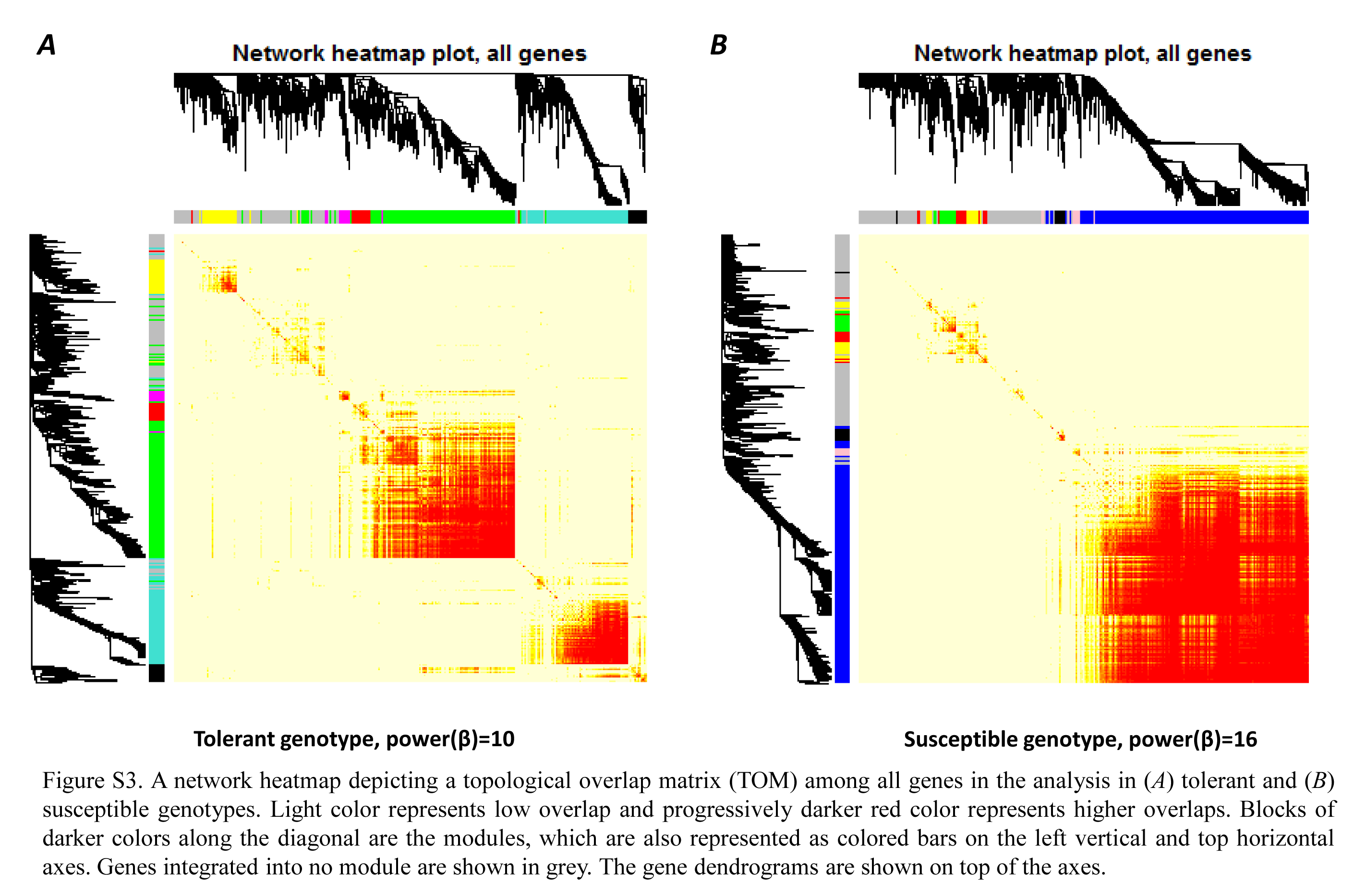

Supplement: Figure S3 — A network heatmap depicting a topological overlap matrix (TOM) among all genes in the analysis in (A) tolerant and (B) susceptible genotypes. Light color represents low overlap and progressively darker red color represents higher overlaps. Blocks of darker colors along the diagonal are the modules, which are also represented as colored bars on the left vertical and top horizontal axes. Genes integrated into no module are shown in grey. The gene dendrograms are shown on top of the axes. (TIF) [file pone.0070433.s003.tif]
